# Opinions of the Midwives about Enabling Factors of Skin-To-Skin Contact Immediately after Birth: A Descriptive Study 

**Published:** 2014-09

**Authors:** Fatemeh Nahidi, Sedigheh Sadat Tavafian, Mohammad Haidarzade, Ebrahim Hajizadeh

**Affiliations:** 1 Tarbiat Modares University, Tehran Iran; 2 Department of paediatrics, Tabriz Medical Sciences University, Tabriz, Iran; 3 Department of Biostatistics, Medical Sciences Faculty, Tarbiat Modares University, Tehran, Iran

**Keywords:** PRCEDE-PROCEED Model, Enabling Factors, Hospitals, Midwives, Skin-to-Skin Contact

## Abstract

**Objective:** Despite the benefits of mother-newborn skin-to-skin contact (SSC) immediately after birth, the process has not been universally implemented as routine care for healthy term neonates. The purpose of this study was to determine opinions of the midwives about enabling factors of SSC immediately after birth in Tehran hospitals in 2012- 2013.

**Materials and methods:** This study has been conducted in a descriptive method based on PRCEDE-PROCEED model. The samples were 292 midwives from 18 hospitals. We used stratified and then simple random sampling. In this study midwives were working at delivery room; deliveries were conducted by midwives or they were in charge of the newborn immediately after birth. Data collection instrument was a self developed questionnaire concerning the enabling factors in the SSC. We applied face and content validity ratio (CVR), content validity index (CVI) and item impact method for the instrument's validity and Cronbach's Alpha for reliability. Finally, data were analyzed and interpreted using spss-18 through descriptive statistics.

**Results:** The results show that 90.4% of the midwives believed in necessity of a plan, 96.2% believed that good services should be provided to mothers, and 97.9%, 85.3% and 93.8% of them believed there is a need for private space, essential facilities and essential equipment for skin contact process; with Cronbach's Alpha of 0.731, 0.551, 0.501, and 0.600 respectively.

**Conclusion:** Most of the midwives believed that enabling factors concerning the successful SSC are effective. We suggest further studies on other enabling factors effective in SSC from the view point of midwives, gynaecologists and caregivers.

## Introduction

The contact between skins of the mother and the newborn immediately after birth is a form of appropriate care for a healthy, term neonate (1). Findings of studies over the last 25 years suggest that the first hour after birth is a critical time for bonding between mother and child, when both are ready for a coordinated reciprocal interaction (2- 7). Instinctive nourishing behaviours, including seeking and breastfeeding, start in this time (8). Another advantage is the improvement in mother’s ability for caring for her child (9), the long term positive impact of attachment behaviours (10- 12), reduced stress of mother and newborn, finding ways to counter stress (10), regulation of breathing, heartbeat, and body temperature of the newborn, calm sleep, shortened interval between delivery and breastfeeding, success in first breastfeeding, elongation of breastfeeding period (13), regulation of neonatal blood sugar level and reduced child cries (14) and reduced behavioural problems (12). Despite the abundance of evidence suggesting the positive impact of immediate mother and neonate skin contact, it has not been adopted as a universal item of post-delivery care for healthy term children (15). SSC is a simple and cost-effective method for improving post-delivery care, encouraging exclusive breastfeeding (12) by midwives. Midwifery is an important occupation in the field of labour and social health, and providing obstetrical counsel and services is a responsibility of midwives. 

According to an unofficial report by the databank of Iranian Ministry of Health, estimates the number of midwifery graduates to be 55 thousand individuals by 2012 (16). The midwife is the first person to contact the neonate after birth, it is crucial to modify the attitude and behaviours of midwives as the first caregiver of children. Despite the evidences suggesting the positive impact of the immediate mother and newborn SSC, it has not yet been adopted as an Iranian item in post-delivery care for healthy children and no study has explained the reasons.

Therefore, it is necessary to identify the factors associated with SSC immediately after birth. The purpose of this study was to determine opinions of the labour working midwives regarding to enabling factors of SSC immediately after birth in Tehran hospitals in 2012-2013. We were using Enabling factors of the Precede-Proceed model that provides a pattern for planning developed by Green and Kreuter in 1970 (16). Enabling factors is pave the way to behavioural or environmental modifications which allow for realization of a motivation or environmental policy and affect the person’s behaviour directly or indirectly via environmental factors, such as regulations, laws, health plans, availability of services, access to necessary resources, and having the skill (17, 18).

## Results

In this study, 292 midwives with the following conditions were recruited: mostly at the age of 40-49 years (34.9%), with the mean of 36.06±8.72; work experience of 10-19 years (36%), with the mean of 11.07±8.29 and with the last child of 1-9 years (46.6%); the demographic and obstetric characteristics of the midwives are presented in (table 1).

In this study, statements with CVR values of equal to/ higher than 0.40 and with CVI scores of equal to/ higher than 0.79 were recorded. The minimum and maximum factor impact scores were recorded as 3.57 and 4.67, respectively. 

**Table 1:** Distribution of the demographic and obstetric characteristics of the delivery room working midwives (n=292)

	**n (%)**	
Type of Hospital	Educational	125 (42.8)
	Organization of Social Security	80 (27.4)
	Private	87 (29.8)
Employment status	Official	124 (42.5)
	Contractual	29 (9.9)
	By project	92 (31.5)
	Mandatory service	47 (16.1)
Marital status	Married	180 (61.6)
	Single	111 (38.1)
	Divorced	1 (0.3)
Degree in midwifery	Associates Degree	14 (4.8)
	Bachelor Degree	257 (88.0)
	Master Degree	21 (7.2)
Midwife’s job interest	Yes	231 (79.1)
	No	61 (20.9)
Number of gravidity	No	144 (49.3)
	Once	65 (22.2)
	Twice	76 (26)
	Three times	7 (2.3)
Number of children	No	144 (49.3)
	One	64 (21.9)
	Two	75 (25.6)
	Three	9 (3.1)

**Table 2:** Distribution of the delivery working midwives' responses to the existence of a plan for SSC immediately after birth

**Questions about the existence of a plan for SSC immediately after birth** ** n=292**	**Yes ** **n (%)**	**Don’t know** **n (%)**	**No** **n (%)**
Presence of a supportive program in the ministry improves SSC	249 (85.3)	30 (10.3)	13 (4.5)
Presence of a skill teaching program in hospital improves SSC	262 (89.7)	24 (8.2)	6 (2.1)

Score < 33= 8 (2.7%), Score 33- 66= 20 (6.8%), Score > 66= 264 (90.4%); Cronbach's Alpha= 0.731

**Table 3:** Distribution of the delivery working midwives' responses to the questions about the services provided for mothers concerning the SSC immediately after birth

**Questions about the Services provided for mothers in delivery room** **n= 292**	**Yes ** **n (%)**	**Don’t know ** **n (%)**	**No ** **n (%)**
Physiologic delivery has a positive impact on skin-to-skin contact	265 (90.8)	20 (6.8)	7 (2.4)
Educating mother during pregnancy improves skin-to-skin contact	281 (96.2)	10 (3.4)	1 (0.3)
Encouraging the mother to have skin contact in labour room will improve SSC	282 (96.6)	7 (2.4)	3 (1)
Relief drug administration during delivery interferes in SSC	167 (57.2)	76 (26)	49 (16.8)
Educating companion improves skin-to-skin contact	260 (89)	24 (8.3)	8 (2.7)
Educating the parent before pregnancy improves skin-to-skin contact	264 (90.5)	20 (6.8)	8 (2.7)
Collaboration of the labor-supporting team improves skin-to-skin contact	283 (96.9)	8 (2.7)	1 (0.3)

Score < 33= 1(0.3%), Score 33- 66= 10 (3.4%), Score > 66= 281(96.2%); Cronbach's Alpha= 0.551

**Table 4:** Distribution of the delivery working midwives' responses to the questions the essential facilities for SSC immediately after birth

**Questions about the essential facilities for SSC immediately after birth** **n= 292**	**Yes ** **n (%)**	**Don’t know ** **n (%)**	**No ** **n (%)**
Availability of adequate human resources in labor room improves SSC	276 (94.5)	7 (2.4)	9 (3.1)
Presence of a midwife to take care of the newborn affects skin contact.	283 (96.9)	7 (2.4)	2 (0.7)
Presence of educated companion in the labor room improves SSC	241 (82.5)	37 (12.7)	14 (4.8)

Score < 33= 1(0.3%), Score 33- 66= 5 (1.7%), Score > 66= 286 (97.9%); Cronbach's Alpha= 0. 501

**Table 5:** Distribution of the delivery working midwives' responses to the questions about the essential equipment concerning the SSC immediately after birth

**Question about the essential equipment for SSC immediately after birth** **n= 292**	**Yes** **n (%)**	**Don’t know** **n (%)**	**No** **n (%)**
A suitable delivery bed affects the skin contact	249 (82.5)	37 (12.7)	14 (4.8)

Score < 33= 23 (7.9%), Score 33- 66= 20 (6.8%), Score > 66= 249 (85.3%); Cronbach's Alpha= 0.64

The midwives' opinions about the enabling factors concerning the SSC between mother and newborn immediately after delivery were classified in five categories (tables 2- 6). As you see in table 2 most of the midwives who work in delivery room answered "yes" to the questions concerning the existence of a plan for SSC immediately after birth. The results show that 90.4% of the midwives believed in we necessity of a plan for skin contact.

Most of the interviewees answered "yes" to the questions about type of service provided to mother. As you see in the (table 3); 96.2% of them believed that good services provided to mothers may encourage them to conduct the skin contact process.

Concerning the essential facilities for SSC immediately after birth, the highest percent of answers was "yes". As you see in (table 4), 97.9 percent of interviewees believed in necessity of the essential facilities for skin contact.

Concerning the midwives' opinions about the essential equipment for SSC immediately after birth, most of them answered "yes". As you see in (table 5), 85.3% of them believed in necessity of the essential equipment for skin contact.

Concerning the midwives' opinions about the suitable space for SSC immediately after birth, most of them answered "yes". As you see in (table 6), the idea of 93.8% of the interviewees was" we need essential private space for skin contact process".

Finally, the instrument of enabling factors, with 16 questions and five sections was evaluated as acceptable reliability, with the Cronbach's alpha of 0.714.

**Table 6:** Distribution of the delivery working midwives' response to the questions about the suitable space for SSC immediately after birth

**Questions about the suitable space for SSC immediately after birth** **n=292**	**Yes ** **n (%)**	**Don’t know ** **n (%)**	**No ** **n (%)**
The temperature of the labor room affects skin contact.	259 (88.7)	21 (7.2)	12 (4.1)
Availability of private space during labor affects skin contact.	240 (82.7)	30 (10.3)	22 (7.5)
Presence of an appropriate space in the operation room affects skin contact.	262 (89.7)	18 (6.2)	12 (4.1)

Score < 33= 3 (1%), Score 33- 66= 15 (5.1%), Score > 66= 274 (93.8%); Cronbach's Alpha= 0.600

Most of midwives who work in delivery rooms answered "yes" to questions about the enabling factors of SSC immediately after birth.

Data analysis showed that 97.3% of delivery working midwives assumed good scores in this section. It means that most of the midwives agreed to enabling factors concerning the skin to skin contact are effective in the process and just 2.7% of them had no idea.

## Discussion

In our study on the delivery room working midwives' opinions, the enabling factors consisted of five sections or sub-structures. Most of them believed that the enabling factors concerning the skin the skin contact between mother and newborn immediately after delivery influence the process success.

The findings of the first sub-structure in our study related to existence of skin contact plan. Most of midwives agreed with necessity of such plans. This section was in conformity with opinions of Glanz (2008) and Lawrence (2005) who assumed health regulations, laws and plans as prerequisites of behaviour changes. Perhaps, well-structured plans along with obligatory circulars on SSC make midwives conduct the process more accurately.

The second sub-structure related to the service type. Most of the subjects believed the high quality services encourage mothers to conduct SSC which is again confirmed by earlier studies including those of Glanz (2008) and Lawrence (2005). They believed that service access facilitates the behavioural changes (17, 18). In this section, midwifes believed that delivery type, mothers training, mothers encouragement, painless delivery, training with cooperation of delivery support team may enhance behavioural changes and they must be considered in successful SSC by midwives, patients and the patients' attendances. Therefore, it is felt that realization of successful SSC requires a widespread and comprehensive planning.

The third sub-structure related to necessary SSC sources received a good score, again in conformity with the fin dings of Glanz (2008) and Lawrence (2005) as the required sources are crucial in behavior change (17, 18). If the number of service providers are enough and at standard level, SSC will be successful.

Concerning the fourth sub-structure of the present study i.e. the necessary facilities, the findings revealed that the midwives assumed the facilities including proper delivery bed pave the way for successful SSC. The point is confirmed by Glanz and Lawrence (17, 18). Generally, appropriate facilities are among the behaviour change antecedents which facilitate standard and successful SSC.

And the fifth sub-structure related to the necessary space for SSC received a good score according to the midwives. They believed that the suitable temperature and private space may enhance SSC. The finding is conformity with those of Glanz and Lawrence; (17, 18). Because environmental factors affect the behavioural change directly or indirectly as well as on the care conducts for the first time. It must be noted that there is no similar study in the literature or at least we did not find any similar study to be used in this research. Therefore, this is the first study on enabling factors effects from the view point of midwives as service providers and the first individuals who are in contact with newborns. 

The working midwives believed that enabling factors concerning the successful SSC are effective. Among the enabling factors we studied plans, service type, necessary sources, facilities and suitable delivery space. Providing these factors may overcome SSC barriers and helps the health team in this regard. The author suggests further studies on other enabling factors which are effective in SSC from the view point of midwives. Then the findings must be compared to those of other countries' research aiming at determining the shortages and weak points of SSC in a more precise way. 

## References

[1] Moore ER, Anderson GC, Bergman N, Dowswell T (2012). Early skin-to-skin contact for mothers and their healthy newborn infants. Cochrane Database Syst Rev.

[2] Widstrom AM, Ransjo-Arvidson AB, Christensson K, Matthiesen AS, Winberg J, Uvnas-Moberg K (1987). Gastric suction in healthy newborn infants: Effects on circulation and developing feeding behaviour. Acta Paediatrica Scandinavica.

[3] Karl, D. (2004). Using principles of newborn state organization to facilitate breastfeeding. MCN American Journal of Maternal Child Nursing.

[4] Sloan NL, Camacho LW, Rojas EP, Stern C (1994). Kangaroo mother method: randomised controlled trial of an alternative method of care for stabilized low-birthweight infants. Lancet.

[5] Bystrova K, Widstrom AM, Matthiesen AS, Ransjö-Arvidson AB, Welles-Nyström B, Wassberg C (2003). Skin-to-skin contact may reduce negative consequences of “the stress of being born”: a study on temperature in newborn infants, subjected to different ward routines in St. Petersburg. Acta Paediatr.

[6] Ransjo-Arvidson AB, Matthiesen AS, Lilja G, Nissen E, Widström AM, Uvnas-Moberg K (2001). Maternal analgesia during labor disturbs newborn behavior: effects on breastfeeding, temperature, and crying. Birth.

[7] Charpak N, Ruiz Pelaz JG, Charpak Y (1994). Rey-Martinez kangaroo mother program: an alternative way of caring for low birth weight infants? One year mortality in a two cohort study. Pediatrics.

[8] Larry Gray, Lisa Watt, Elliott M. Blass (2000). Skin-to-Skin Contact Is Analgesic in Healthy Newborns. Pediatrics.

[9] Michelsson K, Christensson K, Rothgänger H, Winberg J (1996). Crying in separated and non-separated newborns: sound spectrographic analysis. Acta Paediatr.

[10] Ferber SG, Makhoul IR (2004). The effect of skin-to-skin contact (kangaroo care) shortly after birth on the neurobehavioral responses of the term newborn: a randomized, controlled trial. Pediatrics.

[11] Klaus M (1998). Mother and Infant: Early Emotional Ties. Pediatrics.

[12] Klaus MH, Jerauld R, Kreger NC, McAlpine W, Steffa M, Kennel JH (1972). Maternal attachment. Importance of the first post-partum days. N Engl J Med.

[13] Widstrom AM, Lilja G, Aaltomaa-Michalias P, Dahllof A, Lintula M, Nissen E (2011). Newborn behaviour to locate the breast when skin-to-skin: a possible method for enabling early self-regulation. Acta Paediatrica.

[14] Baby-Friendly USA (2003). Overcoming Barriers to Implementing the Ten Steps to Successful Breast feeding Massachusetts.

[15] Gartner L, Morton J, Lawrence R, Naylor A, O’Hare, D, Schanler, R, American Academy of Pediatrics policy statement (2005). Breastfeeding and the use of human milk. Pediatrics.

[16] Robert TC ( 2005). Theory at a glance: a guide for health promotion practice.

[17] Glanz K, Rimer B, Viswanath K (2008). Health Behavior and Health Education Theory, Research, and Practice. Using the PRECEDE-PROCEED Model. 3 edition Jossey-Bass.

[18] Lawrence W. Green, Marshall W, Kreuter (2005). Health Program Planning: An educational and ecological approach.

[19] Munhall PL (2006). Nursing Research: A Qualitative Perspective.

[20] Wood L, Haberd G (2002). Nursing research: methods, clinical appraisal, and utilization.

[21] Abedi HA, Ravani Pour M, Karimollahi M, Yousefi H (2007). Qualitative research methods in nursing.

[22] Hajizadeh E, Asghari M (2011). Statistical Methods and Analyses in Health and Biosciences, A Research Methodological Approach, Using SPSS Practical guide.

[23] Hyrkas K, Appelqvist SK, Oksa L (2003). Validating an instrument for clinical supervision using an expert panel. Int J Nurs Stud.

[24] Lawshe CH (1975). A quantitative approach to content validity. Personnel Psychology.

[25] Waltz CF, Bausell RB (1983). Nursing research: Decision statistics and computer analysis.

[26] Juniper EF, Guyott GH, Streiner DL (1997). Clinical impact versus factor analysis for quality of life questionnaire construction. Journal of Clinical Epidemiology.

[27] Lacasse Y, Godbout C, Series F (2002). Health related quality of life in obstructive sleep apnea. European Respiratory Journal.

[28] Broder HL, McGrth C, Cisneros GJ (2007). E Questionnaire development: face validity and item impact testing of the child oral Heath impact profile. Community Dent Oral Epidemiol.

